# Did you hear that? The role of stimulus similarity and uncertainty in auditory change deafness

**DOI:** 10.3389/fpsyg.2014.01125

**Published:** 2014-10-02

**Authors:** Kelly Dickerson, Jeremy R. Gaston

**Affiliations:** Human Research and Engineering Directorate, US Army Research LaboratoryAberdeen Proving Ground, MD, USA

**Keywords:** change deafness, similarity effects, uncertainty, informational masking, environmental sound perception, complex sound perception

## Abstract

Change deafness, the auditory analog to change blindness, occurs when salient, and behaviorally relevant changes to sound sources are missed. Missing significant changes in the environment can have serious consequences, however, this effect, has remained little more than a lab phenomenon and a party trick. It is only recently that researchers have begun to explore the nature of these profound errors in change perception. Despite a wealth of examples of the change blindness phenomenon, work on change deafness remains fairly limited. The purpose of the current paper is to review the state of the literature on change deafness and propose an explanation of change deafness that relies on factors related to stimulus information rather than attentional or memory limits. To achieve this, work on across several auditory research domains, including environmental sound classification, informational masking, and change deafness are synthesized to present a unified perspective on the perception of change errors in complex, dynamic sound environments. We hope to extend previous research by describing how it may be possible to predict specific patters of change perception errors based on varying degrees of similarity in stimulus features and uncertainty about which stimuli and features are important for a given perceptual decision.

Everyday listening environments are complex, containing many simultaneously occurring sounds that can vary significantly along a wide range of dimensions. Despite substantial, naturally occurring variability, listeners are typically able to extract source-relevant information to detect, localize, and identify meaningful sound source changes in their environment. These processes can be considered together under the function of *auditory scene analysis*, in which individual sound features are segmented or bound into coherent sound “objects.” An important outcome of a successful auditory scene analysis is the ability to notice behaviorally meaningful changes in complex environments (Bregman, 1994; Snyder et al., 2012).

When these processes fail it can have potentially disastrous consequences. Listeners may not notice their own cellular ring tone, or may not hear passing vehicles. Take for example, a busy construction site, where numerous competing sound sources can overlap with varying degrees of sensory/perceptual and semantic-level similarity. Additionally, variability in the number and spatial distribution of sound sources can lead to uncertainty about what sound sources are important. If the sound of a truck backing up goes unnoticed because of the presence of other similar sound sources (e.g., loaders, generators, other trucks), serious injury may occur. For this reason, backup alarms were added to construction vehicles to help distinguish them from other workplace sounds. Still, accidents can occur even when the alarm is very distinct (e.g., Vaillancourt et al., 2013). This is a real world example of the laboratory phenomenon of *change deafness*—where behaviorally meaningful changes within complex auditory scenes are often missed (Gregg and Samuel, 2008). This phenomenon is the auditory analog to visual change blindness (i.e., failures to notice large changes in a visual scene; e.g., Simons and Rensink, 2005).

The first study to document *change deafness* used a classic dichotic listening task; independent streams of speech were presented to each of a listener's ears, and the listener had to selectively attend to one stream (Cherry, 1953). Across a number of conditions, the stream in the unattended ear was altered, and listeners were asked questions about the nature of the changes. Global changes, such as a change from speech to a tone or from a male to female voice, were always detected. But, listeners failed to notice local changes (e.g., language changed from English to German). Contemporary examples of change deafness for speech (Vitevitch, 2003; Sinnett et al., 2006; Fenn et al., 2011), music (Agres and Krumhansl, 2008), and environmental sounds (Eramudugolla et al., 2005; Pavani and Turatto, 2008; McAnally et al., 2010; Gregg and Snyder, 2012) are emerging, however, the literature remains fairly limited. Recent change deafness studies have implemented the *one-shot paradigm*, similar in sprit to the *flicker paradigm* used in change blindness studies, which is essentially a same-different task. In this task a scene is presented for a short duration, then, after a brief inter-stimulus-interval (ISI), a second scene is presented. The second scene is either the same as the first or contains a source change and listeners are asked if a change occurred across the two presentations. Change deafness occurs with error rates around 30%; this level is common across a number of studies (e.g., Gregg and Samuel, 2008; Snyder and Gregg, 2011; Vitevitch and Donoso, 2011; Backer and Alain, 2012).

## Factors mediating change deafness

Studies of change deafness have traveled the well-worn path set forth by researchers examining change blindness. Spatial separation, delay interval, scene size, category membership, pre- and post-change cueing, and familiarity, all influence the likelihood of change perception errors in both visual (e.g., Simons and Rensink, 2005) and auditory modalities (e.g., Snyder and Gregg, 2011). While such a broad and extensive map of the factors that influence performance is helpful, it does little to inform the discussion of the potential underlying mechanisms. To uncover the mechanisms responsible for errors in auditory change perception, a unifying construct, within which factors such as cueing, familiarity, category, and spatiotemporal effects would fit, would be beneficial. In the following sections, we discuss how stimulus *similarity* and *uncertainty* relate to the patterns of errors observed in change deafness and other related auditory phenomenon, and how these factors together may explain the nature of perceptual errors in complex environments.

### Similarity

Similarity, the likeness or featureal overlap between sources, exerts a strong influence in a number of auditory tasks, from low-level sensory-perceptual to higher-level cognitive (see Leech et al., 2009). In traditional psychophysical tasks, stimuli vary along a single dimension, thus defining similarity among items is a relatively straightforward operation. For complex sounds, such as everyday environmental sounds, the definition of similarity requires an account of variability across multiple dimensions, making the definitional problem vastly more difficult. One approach to defining similarity that has been adopted in environmental sound research (e.g., Ballas, 1993; Gygi et al., 2007 has been to generate estimates of perceptual space based on subjective similarity ratings. Typical estimates are based on multidimensional scaling (MDS) analyses, and the degree of feature overlap among sources is represented as a spatial map (Young and Hamer, 1987) and can be used to quantify similarity among stimuli. This approach is sufficiently flexible to quantify similarity across various dimensions (whether well-defined or not) depending on specifically what is asked of a listener (more on MDS estimates in a later section). Similarity characterized in this way is well-suited to studies of change deafness, as it enables examination of specific subsets of sensory/perceptual and semantic-level factors. Gregg and Samuel (2008, 2009) demonstrated that both sensory/perceptual and semantic-level similarity is linked to the magnitude of change perception errors. In that study, sensory/perceptual similarity was manipulated along dimensions of pitch and harmonicity, and semantic similarity was manipulated based on experimenter-defined category membership (within- vs. between-category changes). Change perception was most accurate when the source of the change was distinct from the background at both semantic and sensory/perceptual levels. Gregg and Snyder (2012) reported similar behavioral results and extended previous reports by demonstrating increased amplitudes in both early (i.e., N100) and late (i.e., P300) components of event-related scalp potentials (ERPs) for detected changes, suggesting a reduced cortical activation during change deafness. More recently, Gregg et al. (2014) reported enhanced P300 activity for detected changes (see also Puschmann et al., 2013). Together, these results demonstrate that both sensory/perceptual and semantic-level similarity are important, and that the degree of overlap among individual features or sound objects should be directly related to performance. However, as noted above, this notion can be difficult to test since quantifying systematic differences for stimuli that vary along many dimensions is challenging, especially for complex environmental sounds (See McDermott et al., 2009, 2013 for related discussion).

### Uncertainty

In any experimental task, stimulus uncertainty (e.g., number of competing sources, varying spatial position) can impact listener performance, but what seems to matter most is the difference between what a listener hears, and what they expect to hear (Durlach et al., 2003). Like similarity, psychophysical definitions of uncertainty can be relatively straightforward, when uncertainty is limited to single, or few dimensions. Even for complex environmental sources this may be true, but not necessarily when embedded in real-world contexts where there may be numerous sources of uncertainty. Uncertainty effects have been demonstrated across a number of auditory phenomena ranging from low-level detection masking, to mid-, and higher-level effects such as auditory search, change deafness or the cocktail party problem. Variability in the target or background (contextual) stimuli (occurring within or across-trials) and the magnitude of these variations can lead to a range from minimal to high uncertainty. A good example of minimal uncertainty is the *auditory detection-masking paradigm* (e.g., Fletcher, 1940; see Moore, 2012) where detection of a well-defined and known pure-tone target is measured as a function of the bandwidth of a white noise masker centered at the target frequency. Here, there is essentially no uncertainty associated with the target, and only minimal uncertainty about the masker, leading to precise, and stable masking thresholds over time. Because this form of detection masking is due to interactions at the auditory periphery, it is sometimes referred to as *energetic masking* (Kidd et al., 2008).

Unlike *energetic masking, informational masking* (IM) is highly dependent on the degree of stimulus uncertainty, and is thought to occur at more central levels of processing, and is related to multiple stages of processing. IM is related to a number of perceptual and cognitive constructs, such as attention, memory, and perceptual grouping (Kidd et al., 2008; Best et al., 2012). In the basic IM paradigm, a target tone is presented simultaneously with a varying number of contextual “masking” tones. The frequency difference between the target and the competing sounds is always at least an equivalent rectangular bandwidth (ERB: estimate of the size of the auditory filter; see Moore, 2012) from any of the masking sounds to prevent *energetic masking*. The general result is that target detection thresholds increase as a function of the number of masking tones. This increase in target detection thresholds is monotonic up to a critical masker density, with a reduction or asymptote beyond this critical limit (e.g., Lutfi et al., 2003).

In typical IM paradigms, uncertainty is primarily driven by spectral or temporal variation in contextual elements; uncertainty about the target is fairly minimal. This is generally a matter of necessity, since targets typically need to be well-defined and well-known for listeners to make perceptual decisions. In the real world, as in *change deafness*, not only is the context highly uncertain due to the presentation of multiple sound sources, but so is the target. This is because the target can be any one of the sounds presented on any given trial. Moreover, the change sound can be drawn from any of the entire set of sounds in the input distribution, thus introducing substantial across-trial uncertainty. Similar to IM, in *change deafness*, the magnitude of errors seems to be related to the magnitude of stimulus uncertainty. For example, Eramudugolla et al. (2005) found that reducing uncertainty, by cueing the identity of the change item and spatially separating sounds in a scene could significantly reduce errors (see also Backer and Alain, 2012 for discussion on pre- vs. post-change cueing). However, even with substantial spatial separation, sound localization errors systematically increase with the number of background sources (Simpson et al., 2007).

## A framework for understanding similarity and uncertainty effects

As discussed in the preceding sections, the stimulus factors of similarity and uncertainty are important for a number of auditory phenomena, including *change deafness*. In traditional psychophysical approaches, it is common to map perception of changes across individual stimulus dimensions, and, because of this, it is common practice to treat the contributions of similarity and uncertainty as independent. For example, measurement of the frequency difference threshold between two tones (e.g., Moore, 2012) can be operationally defined as the measurement of the similarity between those tones. If the target stimulus remains fixed, the only (minimal) uncertainty is due to the change in comparison tone frequency. By contrast, in the typical simultaneous IM paradigm, the target is always dissimilar from the contextual background tones, and changes in the detection thresholds are the result of changes in the number of background tones. In more complex IM designs, the negative effects of high uncertainty can be mitigated when the similarity between target and contextual tones is further reduced, thus resulting in reduced detection thresholds (e.g., Kidd et al., 1994; Neff, 1995; Durlach et al., 2003).

The interaction of similarity and uncertainty should be even more pronounced for complex listening tasks using environmental sounds that are often multidimensional and dynamic in nature. This seems to be the case for the *change deafness* phenomenon where there is clearly substantial across-trial uncertainty about both the identity of the changed source and the contextual background in which it is presented, all in addition to varying levels of within-trial similarity among sources.

Understanding the relationship between the stimulus factors of similarity and uncertainty and how they affect performance, can provide a predictive account of *change deafness*. A systematic mapping of the relationships of stimulus uncertainty, while still complicated, is probably the most straightforward and would require independent control of uncertainty for target and contextual sounds, both within and across-trials. A systematic mapping of similarity presents a more difficult proposition, especially given potentially suprathreshold differences across many dimensions.

As introduced earlier, one method to define similarity could be borrowed from approaches used in the environmental sound perception literature where listener perceptual space for a particular set of sounds is estimated based on listener-generated similarity ratings through the application of multivariate statistical techniques such as MDS (e.g., Gygi et al., 2007; Gaston and Letowski, 2012). By themselves, listener ratings cannot be directly tied to specific sensory/perceptual or semantic-level dimensions. Rather, they reflect similarity based on all of the perceived differences between stimulus items in a set. A good example of this approach comes from Gygi et al. (2007), who collected similarity ratings for sound pairs from a set of 50 environmental sounds, and analyzed the ratings using MDS. They then compared the mapping of sound sources in the MDS solution with measured distributions of spectral-temporal acoustic descriptors to relate the physical attributes of the sounds to listener perceptual space. Using this type of approach, similarity can be defined as the Euclidean or “city-block” distances between sound sources in the MDS solution, and thus provides a degree of systematicity in defining differences between stimuli (Young and Hamer, 1987). Additionally, identifying those properties (whether low-level sensory/perceptual or higher-level cognitive/semantic) that are correlated with MDS space can generate hypotheses about the information that may be important in perceptual decisions. There is at least some evidence that there can be good agreement between the organization of MDS space, based on perceived similarity, and the recognition of specific sets of environmental sounds (Gaston and Letowski, 2012). Likewise, in the *change deafness* paradigm, similarity can be defined by the relationship between the change sound and the estimated perceptual distance from contextual sounds. Indeed, Gregg and Samuel (2009) used the results of Gygi et al. (2007) as the basis for selecting sounds differing along the sensory/perceptual dimensions of pitch and harmonicity.

### A path forward

Understanding the perception of environmental sound sources is difficult because of the complexity inherent to this broad class of sounds. In general, as complexity increases, so do potential sources of variability, which may or may not be source-relevant (e.g., Pastore et al., 2008). This complexity, which can be related to important stimulus dimensions or simply noise, can be problematic for traditional psychophysical approaches that seek to map perception as a function of systematic changes in single, well-defined stimulus dimensions. Environmental sounds are rarely uni-dimensional and stimulus differences across- and within-sound classes are rarely systematic. Speech is similarly complex and the relatively good understanding of this sound class is largely based on psychophysical mappings of speech properties (e.g., see Raphael, 2008). However, this mature understanding has had the benefit of more than 75 years of systematic, incremental research. Compared to speech perception, the study of environmental sound perception is relatively new, and supporting research has been limited.

Psychophysical approaches by themselves may be sufficient to make this problem tractable, but would require an incredible amount of time and effort. One alternative is to consider various broad classification metrics that map onto global representations of similarity between sound sources (i.e., MDS space). This approach has the benefit of enabling collection of perceptual similarity data on a large stimulus set relatively quickly. The relationship between this more global representation and both sensory/perceptual and semantic dimensions can drive predictions about information that may be important in differentiating environmental sound classes (e.g., Ballas, 1993; Gygi et al., 2007; Gaston and Letowski, 2012). These relationships would only be based on correlations, but they could ultimately support predictions for targeted psychophysical examinations of potentially relevant stimulus information.

## Concluding comments

The link between stimulus information and performance is important in understanding perception in the real world. *Change deafness* reflects a fundamental limitation to human perceptual experiences, and can help reveal the basic mechanisms underlying perceptual errors in complex listening. These types of complex listening tasks begin to approximate real-world listening conditions, while allowing the use of well-controlled psychophysical techniques common in auditory perception research. In this brief review, a common pattern emerges: errors in change perception can be attributed to the effects of stimulus similarity and uncertainty. Conditions with high informational overlap can increase potential difficulty in effectively allocating attention to feature or feature combinations that are relevant to the listener. It is likely that the patters of errors observed in change deafness are the result of high information load and its impact on allocation of attentional resources (see Lutfi, 1993; Oh and Lutfi, 1998 for examples of informational-attentional interactions in audition; see also Alvarez and Cavanagh, 2004 for visual example). These same patterns are associated with performance in a related phenomenon: the *cocktail party problem*. This is not surprising given the common origin of the phenomena (i.e., Cherry, 1953). *Change deafness* and the *cocktail party problem* demonstrate two extremes of auditory scene analysis. In the one case, high similarity and uncertainty lead to misperceptions, presumably because of a failure to adequately segment the complex scene. In the other case, low similarity and uncertainty create a sort of pop-out effect; the dominant perception is one where the target signal is easily segmented from the background sounds (see Lotto and Holt, 2011). In *change deafness*, it seems that the information reaching memory for encoding is not parsed in a way that enables perception of change, and this may be related to some form of IM influencing early stream segregation processes. The leading view, that change deafness represents a failure to compare between incoming and previously stored information may be part of the story (e.g., Gregg and Samuel, 2009), but what is more likely is that the information being compared is inaccurate or otherwise inaccessible due to informational factors.

### Conflict of interest statement

The authors declare that the research was conducted in the absence of any commercial or financial relationships that could be construed as a potential conflict of interest.
